# Maternal and Fetal Outcomes of Anticoagulation in Pregnant Women With Mechanical Heart Valves

**DOI:** 10.1016/j.jacc.2017.03.605

**Published:** 2017-06-06

**Authors:** Zachary L. Steinberg, Clara P. Dominguez-Islas, Catherine M. Otto, Karen K. Stout, Eric V. Krieger

**Affiliations:** a Division of Cardiology, Department of Medicine, University of Washington School of Medicine, Seattle, Washington; b Medical Research Council Biostatistics Unit, School of Clinical Medicine, University of Cambridge, Cambridge, United Kingdom

**Keywords:** fetal risk, maternal risk, pregnancy, CI, confidence interval, LMWH, low-molecular-weight heparin, MHV, mechanical heart valve, RAR, ratio of averaged risk, UFH, unfractionated heparin, VKA, vitamin K antagonist

## Abstract

**Background:**

Anticoagulation for mechanical heart valves during pregnancy is essential to prevent thromboembolic events. Each regimen has drawbacks with regard to maternal or fetal risk.

**Objectives:**

This meta-analysis sought to estimate and compare the risk of adverse maternal and fetal outcomes in pregnant women with mechanical heart valves who received different methods of anticoagulation.

**Methods:**

Studies were identified using a Medline search including all publications up to June 5, 2016. Study inclusion required reporting of maternal death, thromboembolism, and valve failure, and/or fetal spontaneous abortion, death, and congenital defects in pregnant women treated with any of the following: 1) a vitamin K antagonist (VKA) throughout pregnancy; 2) low-molecular-weight heparin (LMWH) throughout pregnancy; 3) LMWH for the first trimester, followed by a VKA (LMWH and VKA); or 4) unfractionated heparin for the first trimester, followed by a VKA (UFH and VKA).

**Results:**

A total of 800 pregnancies from 18 publications were included. Composite maternal risk was lowest with VKA (5%), compared with LMWH (16%; ratio of averaged risk [RAR]: 3.2; 95% confidence interval [CI]: 1.5 to 7.5), LMWH and VKA (16%; RAR: 3.1; 95% CI: 1.2 to 7.5), or UFH and VKA (16%; RAR: 3.1; 95% CI: 1.5 to 7.1). Composite fetal risk was lowest with LMWH (13%; RAR: 0.3; 95% CI: 0.1 to 0.8), compared with VKA (39%), LMWH and VKA (23%), or UFH and VKA (34%). No significant difference in fetal risk was observed between women taking ≤5 mg daily warfarin and those with an LMWH regimen (RAR: 0.9; 95% CI: 0.3 to 2.4).

**Conclusions:**

VKA treatment was associated with the lowest risk of adverse maternal outcomes, whereas the use of LMWH throughout pregnancy was associated with the lowest risk of adverse fetal outcomes. Fetal risk was similar between women taking ≤5 mg warfarin daily and women treated with LMWH.

Mechanical heart valves (MHVs) are thrombogenic, necessitating long-term anticoagulation to prevent adverse outcomes such as valve thrombosis, stroke, or death. During pregnancy, there is an increase in the production of procoagulant factors, decreased levels of protein S, an acquired protein C resistance, and impaired fibrinolysis leading to an increased risk of thromboembolic events (1), which makes pregnant women especially vulnerable to thrombosis and MHV failure. Vitamin K antagonists (VKAs), such as warfarin, are effective at reducing thromboembolic events and are standard therapy for anticoagulation in the absence of contraindications (2). However, several studies have demonstrated teratogenicity of warfarin during the sixth to ninth weeks of pregnancy 3, 4, 5, and some studies have found a high rate of fetal loss in pregnant women taking warfarin (6). Therefore, many patients and physicians have been reluctant to use a VKA during pregnancy, despite guidelines in the United States and Europe that recommend it for many patients during pregnancy 7, 8.

There has never been a randomized trial comparing different anticoagulation regimens in pregnant women with MHVs, and current guidelines are largely devised on the basis of case series and expert consensus. The most widely cited systematic review (6) was written before the use of low-molecular-weight heparin (LMWH) and included many patients with older-generation ball-in-cage valves, thereby limiting the relevance of this review in contemporary patients. Subsequent systematic reviews and meta-analyses have included women on fixed-dose LMWH, which is now known to be associated with catastrophic valve failure 9, 10.

The goal of this meta-analysis was to estimate the risk of adverse maternal and fetal outcomes among different anticoagulation regimens in a contemporary population of pregnant women with modern MHVs.

## Methods

### Study selection

Studies were identified through a Medline review using the following search terms: “(mechanical valve OR heart disease OR valve replacement OR heart valve) AND pregnancy AND (unfractionated heparin OR heparin OR low molecular weight heparin OR enoxaparin OR warfarin OR low dose warfarin OR oral anticoagulation OR Coumadin OR coumarin OR anticoagulation).” Publications up to June 5, 2016, were included in the search. Each publication was independently adjudicated by 2 of the authors (Z.L.S. and E.V.K.) to determine eligibility for inclusion in the meta-analysis.

Study inclusion required unambiguous reporting of outcomes of interest in pregnant women with MHVs who had anticoagulant therapy with a VKA, LMWH, or unfractionated heparin (UFH). Studies were excluded in the following circumstances: if >10% of reported pregnancies occurred in women with ball-in-cage valves; if fixed doses of either UFH or LMWH were administered; if <5 pregnancies were reported; if pregnancies either were not followed to term or the reported anticoagulation regimen was initiated after the first trimester; if results had previously been published; or if studies were published in a language other than English. Studies reporting outcomes in individuals with mechanical tricuspid or pulmonic valves, in which right-sided valve dysfunction was not specifically reported on, were also excluded out of concern that these valves are at a higher risk for thrombosis and dysfunction 11, 12, 13, 14, 15 that could skew the study results. Attempts were made to include all studies reporting on left-sided MHVs. Results from studies reporting outcomes from both right- and left-sided MHVs, in which valve dysfunction was clearly identified as right sided or left sided, were included. If, however, the position of the dysfunctional valve could not be discerned, the study was excluded. Investigators of each study that met inclusion criteria were contacted if additional data were required.

Four anticoagulation regimens were included in this meta-analysis: 1) VKAs continued throughout the entirety of pregnancy (VKA); 2) dose-adjusted LMWH for the entirety of pregnancy (LMWH); 3) dose-adjusted LMWH for the first trimester, followed by a VKA for the remainder (LMWH and VKA); and 4) dose-adjusted UFH for the first trimester, followed by a VKA for the remainder (UFH and VKA). We also identified women who received low-dose warfarin, defined as ≤5 mg of daily warfarin in individuals able to maintain a therapeutic international normalized ratio (INR).

### Outcomes and definitions

The primary maternal outcome was defined as a composite of maternal death, prosthetic valve failure, and systemic thromboembolism. Prosthetic valve failure was defined as abnormal valve function leading to a clinically meaningful outcome, such as heart failure, arrhythmia, or reoperation. Thromboembolism was defined as any systemic arterial thrombotic event, such as stroke or transient ischemic attack. The primary fetal outcome was defined as a composite of spontaneous abortion, fetal death, and the presence of any congenital defect. The definition of spontaneous abortion was not uniform among the included studies. Spontaneous abortion was defined as any unplanned fetal loss before 20 weeks of gestation in 16 studies 16, 17, 18, 19, 20, 21, 22, 23, 24, 25, 26, 27, 28, 29, 30, 31, 22 weeks in 1 study (32), and 24 weeks in 1 study (33). Fetal death was defined as any unplanned fetal loss at or after 20 weeks of gestation in 16 studies 16, 17, 18, 19, 20, 21, 22, 23, 24, 25, 26, 27, 28, 29, 30, 31, 22 weeks in 1 study (32), and 24 weeks in 1 study (33). To avoid misclassification, we used the definition provided by each individual study. The occurrence of a congenital defect was defined as warfarin embryopathy or any unexpected congenital anomaly for gestational age (excluding patent ductus arteriosus in premature infants). Secondary outcomes included the incidence of maternal death, composite of prosthetic valve failure and systemic thromboembolism, spontaneous abortion, fetal death, and congenital defects.

### Statistical analysis

For the estimation of an average risk of the composite maternal and fetal outcomes, a mixed-effects meta-regression model was fitted on the transformed risks from individual studies, with the different regimens as fixed effects (using VKA, regardless of dose, as the reference) and the different cohorts of women receiving a specific regimen within studies as random effects. For the analysis, a double-arcsine variance-stabilizing transformation 34, 35 was applied to the calculated risks, which allowed the inclusion of cohorts reporting zero events without adding a continuity correction (36). The means of the transformed risks estimated from the meta-regression were then back-transformed (37) to provide an average estimate of maternal and fetal risk for each cohort.

To compare the meta-analytic averaged risks among strategies, an estimate of their ratio was performed and is referred to as the ratio of averaged risks (RAR). The 95% confidence intervals (CIs) for the RARs were obtained using parametric bootstrap, which involved sampling from a multivariate normal distribution assumed for the estimated coefficients from the meta-regression and their covariance matrix and then applying the corresponding back-transformation. Sensitivity analyses were conducted by sampling from a multivariate noncentral Student *t* test distribution. Here we emphasize that the RAR should not be mistaken for an estimate of relative risk because it provides only a measure of the difference among regimens on the risks of adverse events, averaged over different groups of women from nonrandomized observational studies.

In a secondary analysis focusing on adverse fetal outcomes, each alternative regimen (LMWH, LMWH and VKA, and UFH and VKA) was compared with a reference regimen of women receiving low-dose warfarin (≤5 mg daily warfarin) throughout pregnancy. Sensitivity analyses were performed to account for potential misreporting of outcomes. From these analyses, we report the number of unreported events that would have had to occur to change the results of the main analysis significantly.

All analyses were carried out using the statistical software R (R Project for Statistical Computing, Vienna, Austria) (38), and the meta-regression model was fitted using the metafor package (39). We used restricted maximum likelihood for the estimation of the between-cohort variance.

## Results

**Figure 1:**
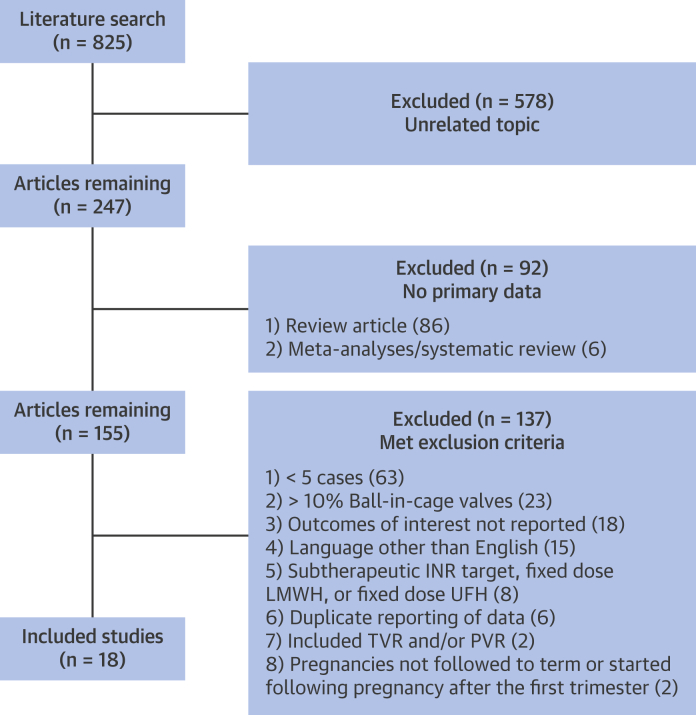
Study Search Flowchart The number and types of studies captured by the search terms. INR = international normalized ratio; LMWH = low-molecular-weight heparin; PVR = pulmonary valve replacement; TVR = tricuspid valve replacement; UFH = unfractionated heparin.

Of the 825 publications identified through the Medline search, 579 were on an unrelated topic, 92 did not include primary patient-related data (e.g., review papers, editorials), and 137 met the exclusion criteria (Figure 1). Eighteen studies, totaling 800 pregnancies between 1974 and 2014, were included in the final analysis 16, 17, 18, 19, 20, 21, 22, 23, 24, 25, 26, 27, 28, 29, 30, 31, 32, 33 (Table 1). Patients in 8 of the studies were followed prospectively. Ten studies reported on maternal and fetal outcomes with a VKA regimen in which most patients were taking warfarin, with a small number of patients taking either acenocoumarol or phenprocoumon. Eight studies reported on maternal and fetal outcomes with an LMWH regimen using enoxaparin and dalteparin. Dose adjustment of LMWH was administered on the basis of anti–factor Xa levels in all 8 studies. Four studies reported on maternal and fetal outcomes with a regimen of LMWH and VKA, and all 4 studies administered LMWH doses on the basis of anti–factor Xa levels. Seven studies reported on maternal and fetal outcomes with a regimen of UFH and VKA; 5 of these studies reported dose-adjusted UFH on the basis of partial thromboplastin time levels, and 2 studies did not report the method for monitoring of UFH. Four studies reported fetal outcomes with a low-dose warfarin regimen. With the exception of 1 study (33), every patient was transitioned to UFH before delivery.

**Table 1:** Characteristics of Included Studies

First Author, Year (Ref. #)	Country of Origin	Regimens	Pregnancies (% of Total)	Dose Adjustment	Valve Types	Pregnancy Date Range
Ayad et al., 2016 (32)	Egypt	VKA UFH + VKA	17 (4) 28 (15)	INR NR + INR	Bileaflet: NR Tilting disk: NR Ball-in-cage: NR	NR
van Hagen et al., 2015 (33)	Multinational	VKA LMWH LMWH + VKA UFH + VKA	38 (8) 17 (20) 31 (72) 47 (25)	INR Anti–factor Xa level Anti–factor Xa level + INR NR + INR	Bileaflet: NR Tilting disk: NR Ball-in-cage: NR	2008–2014
Hassouna and Allam, 2014 (16)	Egypt	Low-dose warfarin	55 (34)	INR	Bileaflet: 100%	1991–2013
Samiei et al., 2012 (19)	Iran	VKA UFH + VKA	43 (9) 10 (5)	INR PTT + INR	Bileaflet: 79% Tilting disk: NR Ball-in-cage: NR	1999–2009
Basude et al., 2012 (17)	United Kingdom	VKA LMWH LMWH + VKA	22 (5) 4 (5) 6 (13)	INR Anti–factor Xa level Anti–factor Xa level + INR	Bileaflet: 94% Tilting disk: 6%	2003–2011
De Santo et al., 2012 (18)	Italy	Low-dose warfarin LMWH	16 (10) 1 (1)	INR Anti–factor Xa level	Bileaflet: 100%	2000–2010
Khamoushi et al., 2011 (20)	Iran	VKA Low-dose warfarin UFH + VKA	38 (8) 29 (18) 11 (6)	INR INR PTT + INR	Bileaflet: NR Tilting disk: NR Ball-in-cage: 0%	2002–2007
Saeed et al., 2011 (21)	South Africa	LMWH	8 (10)	Anti–factor Xa level	Bileaflet: 100%	2007–2009
Quinn et al., 2009 (23)	United Kingdom	LMWH LMWH + VKA	11 (13) 1 (2)	Anti–factor Xa level Anti–factor Xa level + INR	Bileaflet: NR Tilting disk: NR Ball-in-cage: NR	2001–2007
Yinon et al., 2009 (24)	Canada	LMWH	23 (28)	Anti–factor Xa level	Bileaflet: 81% Tilting disk: 14% Ball-in-cage: 5%	1998–2008
Abildgaard et al., 2009 (22)	Norway	LMWH	12 (14)	Anti–factor Xa level	Bileaflet: 92% Tilting disk: 8%	1997–2008
Khamooshi et al., 2007 (25)	Iran	VKA Low dose warfarin UFH + VKA	142 (29) 62 (38) 54 (29)	INR INR PTT + INR	Bileaflet: 50% Tilting disk: 50%	1974–2000
Kim et al., 2007 (26)	Korea	VKA UFH + VKA	5 (1) 18 (10)	INR PTT + INR	Bileaflet: 100%	1990–2005
Descarries et al., 2006 (27)	Canada	LMWH + VKA	5 (12)	Anti–factor Xa level + INR	Bileaflet: 100%	1999–2005
Nassar et al., 2004 (28)	Lebanon	VKA	30 (6)	INR	Bileaflet: 51% Tilting disk: NR Ball-in-cage: NR	1987–2002
Bauersachs and Lindhoff-Last, 2003 (29)	Germany	LMWH	7 (8)	Anti–factor Xa level	Bileaflet: NR Tilting disk: NR Ball-in-cage: NR	1997–2000
Srivastava et al., 2002 (31)	India	VKA	37 (8)	INR	Bileaflet: 34% Tilting disk: 63% Ball-in-cage: 3%	1989–1998
Al-Lawati et al., 2002 (30)	Oman	VKA UFH + VKA	42 (9) 21 (11)	INR PTT + INR	Bileaflet: 33% Tilting disk: 67%	NR

INR = international normalized ratio; LMWH = low–molecular-weight heparin; NR = not reported; PTT = partial thromboplastin time; UFH = unfractionated heparin; VKA = vitamin K antagonist.

Data on the percentage of pregnant women with ball-in-cage valves were missing in 6 studies. Of these, only 1 study included pregnancies before 1998 and accounted for 6% of the total VKA cohort (28). The majority of published outcomes for the VKA, UFH and VKA, and low-dose warfarin regimens originated from Asia, Africa, and the Middle East (85%, 90%, and 76% of pregnancies, respectively). Most published outcomes for the LMWH regimen originated from Europe and North America (79%). Most published outcomes for the LMWH and VKA regimen originated from a multinational study (72%); however, the investigators reported that most patients with this regimen were treated in developed countries (33).

**Figure 2:**
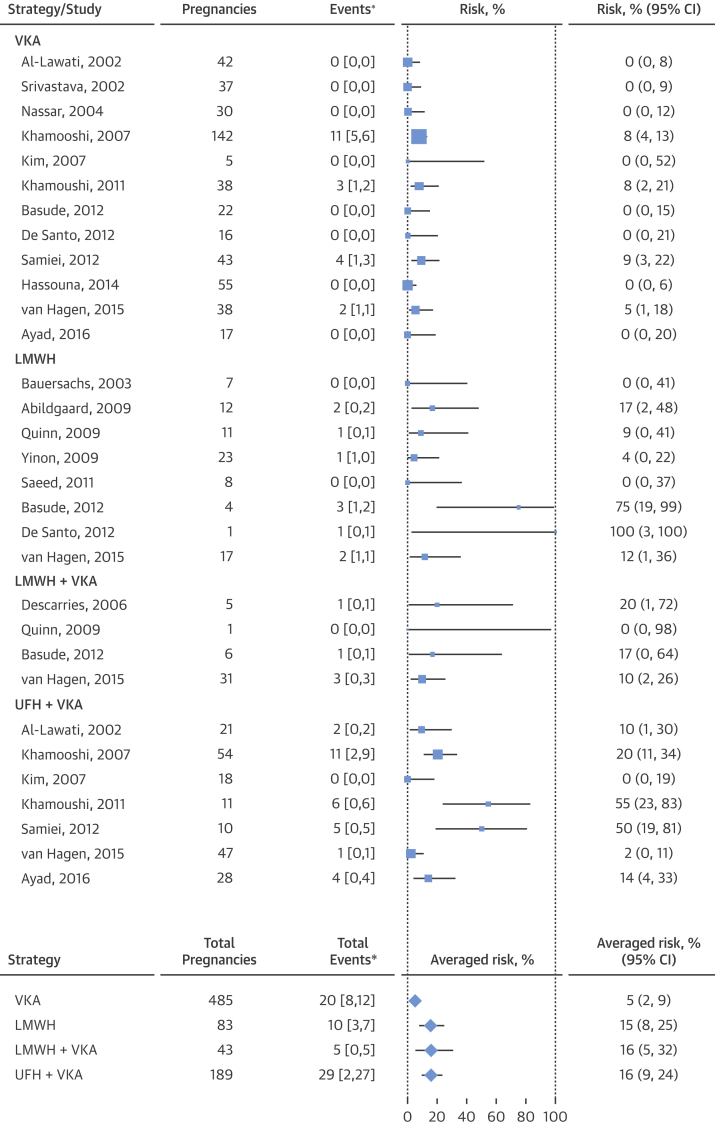
Forest Plot of the Composite Maternal Outcome The individual and composite maternal endpoints as reported by each publication included in the meta-analysis. The forest plot represents an averaged risk of the composite outcome, weighted by study sample size. *Values in brackets are number of deaths, number of prosthetic valve failures or thromboembolisms. CI = confidence interval; VKA = vitamin K antagonist; other abbreviations as in Figure 1.

**Figure 3:**
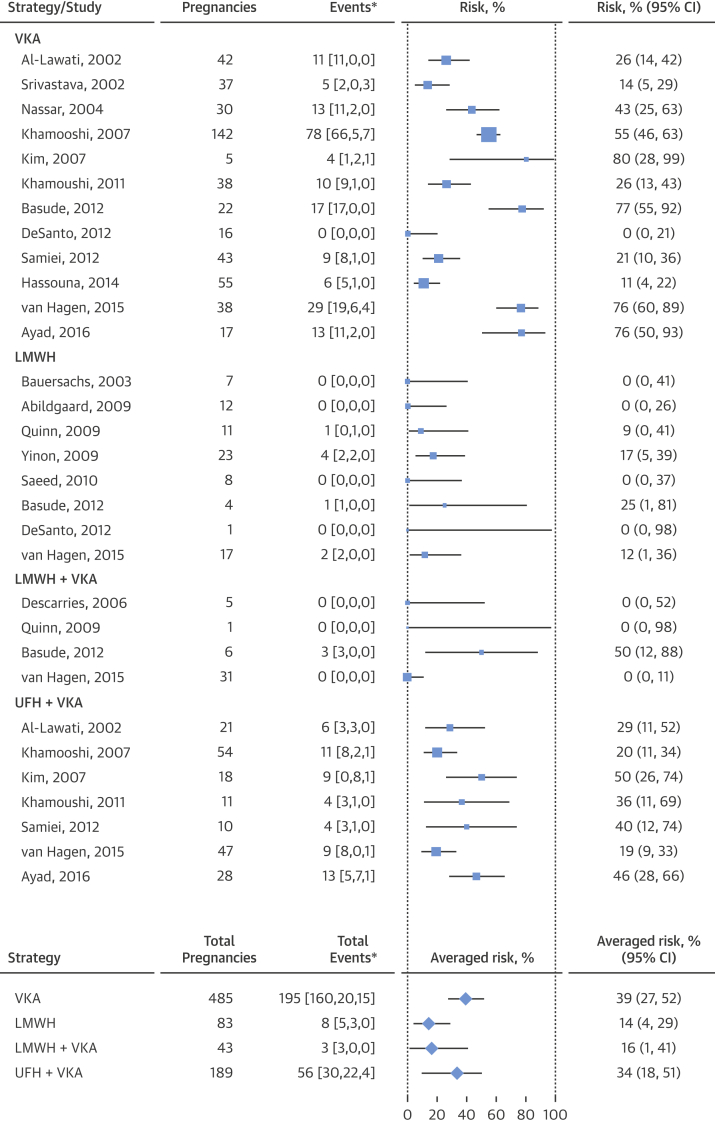
Forest Plot of the Composite Fetal Outcome The individual and composite fetal endpoints as reported by each publication included in the meta-analysis. The forest plot represents an averaged risk of the composite outcome, weighted by study sample size. *Values in brackets are number of spontaneous abortions, number of fetal deaths, number of births with congenital defects. Abbreviations as in Figures 1 and 2.

**Figure 4:**
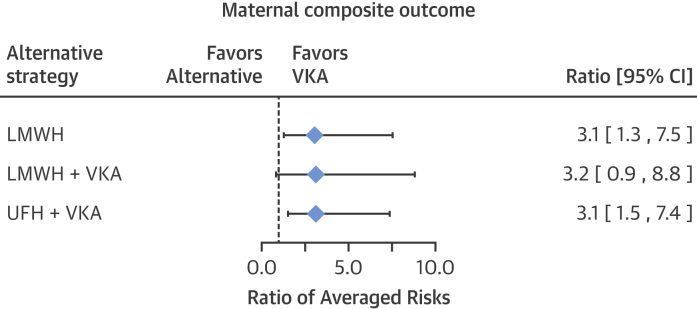
Maternal Composite Outcome Ratio of the meta-analytic averaged risk for the maternal composite outcome between a VKA regimen and each alternative regimen. Abbreviations as in Figures 1 and 2.

The study-specific risks of the composite maternal and fetal outcomes are reported in forest plots (Figures 2 and 3), along with the estimated averaged risks for each regimen, obtained from the mixed-effects meta-regression model on the double-arcsine transformed risks. The estimated averaged risk of the maternal composite outcome was 5.0% (95% CI: 2.5% to 8.5%) for the VKA regimen, 15.5% (95% CI: 7.6% to 25.4%) for the LMWH regimen, 15.9% (95% CI: 4.9% to 31.6%) for the regimen of LMWH and VKA, and 15.8% (95% CI: 9.2% to 23.8%) for the regimen of UFH and VKA. Compared with women who received a VKA regimen, the average risk of an adverse maternal event was significantly higher among women who received an LMWH regimen (RAR: 3.1; 95% CI: 1.3 to 7.5) or a regimen of UFH and VKA (RAR: 3.1; 95% CI: 1.5 to 7.4), with a trend toward significance in women who received a regimen of LMWH and VKA (RAR: 3.2; 95% CI: 0.9 to 8.8) (Figure 4). The number of reported maternal deaths was low across all regimens, and consequently, the incidence of systemic thromboembolism and/or valve failure was largely responsible for the observed differences among groups.

**Figure 5:**
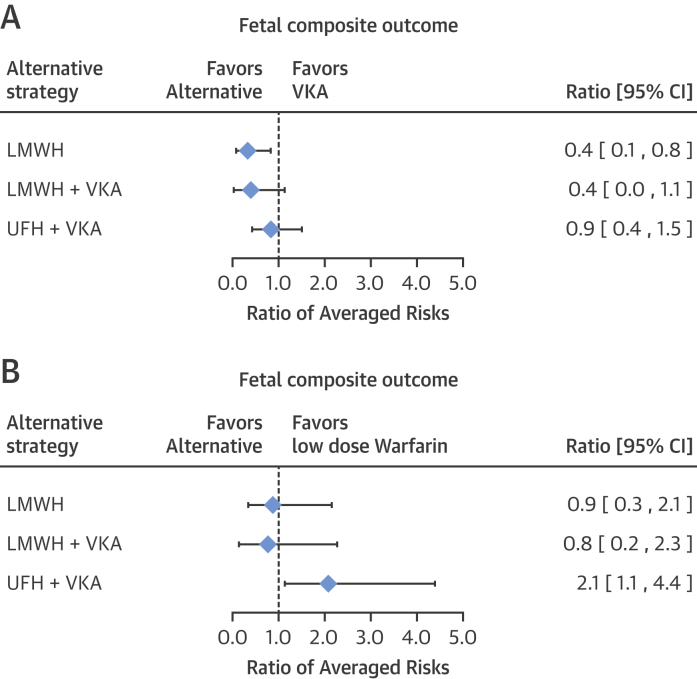
Fetal Composite Outcome **(A)** Ratios of the meta-analytic averaged risk for the fetal composite outcome between a VKA regimen and each alternative regimen. **(B)** Ratio of averaged risk for the fetal composite outcome between a low-dose VKA regimen and the alternative regimens. Abbreviations as in Figures 1 and 2.

The estimated averaged risk of the fetal composite outcome was 39.2% (95% CI: 27.0 to 52.1) for the VKA regimen, 13.9% (95% CI: 3.7 to 29.0) for the LMWH regimen, 16.4% (95% CI: 1.5 to 41.2) for the regimen of LMWH and VKA, and 33.6% (95% CI: 18.4 to 50.8) for the regimen of UFH and VKA. Compared with the VKA regimen, the averaged risk of an adverse fetal event was significantly lower in patients with the LMWH regimen (RAR: 0.4; 95% CI: 0.1 to 0.8) (Figure 5A). No significant difference was observed for the remaining anticoagulation regimens. In a subgroup of individuals taking low-dose warfarin, the estimated averaged risk of the fetal composite outcome was 4.8% (95% CI: 0.0 to 16.9). When the low-dose warfarin regimen was compared with the alternative regimens, no significant differences in fetal risk were observed in comparison with the LMWH regimen (RR: 0.9; 95% CI: 0.3 to 2.1) (Figure 5B).

The number of babies born with congenital defects was unavailable in 1 study reporting outcomes of 3 pregnancies using an LMWH regimen (4.8% of the LMWH cohort) (17), and it was assumed to be zero in the main analysis. The conclusion of the analysis remains unchanged if all 3 babies in this group are assumed to have had congenital defects. Overall, congenital defects and fetal deaths were uncommon events in all groups, with the incidence of spontaneous abortion largely responsible for the observed differences among regimens.

Two studies were excluded because it could not be determined whether outcomes occurred in women with right-sided or left-sided MHVs 40, 41. This decision resulted in the exclusion of 9 pregnancies in women with left-sided MHVs who had a regimen of UFH and VKA and the exclusion of 41 pregnancies in women with left-sided MHVs who had a VKA regimen. Sensitivity analysis demonstrated that the conclusions of the maternal outcomes analysis remained unchanged when assuming that all reported maternal events in the VKA group occurred in individuals with left-sided MHVs and that none of the reported maternal events in the group taking UFH and VKA occurred in individuals with left-sided MHVs (Online Table 1). Similarly, the conclusions of the fetal outcomes analysis remain unchanged regardless of how many of the 6 reported fetal events occurred in individuals with left-sided MHVs who had an anticoagulation regimen of UFH and VKA (Online Table 2).

A significant amount of heterogeneity was present in the estimates of risk among studies. Heterogeneity was estimated to account for 44% (95% CI: 18% to 82%) of the variability of the averaged risk estimates of the maternal composite outcome and for 81% (95% CI: 67% to 90%) of the total variability of the averaged risk estimates of the fetal composite outcome, as displayed by the statistic I^2^ (Online Tables 3 and 4).

## Discussion

On the basis of this contemporary meta-analysis of 800 pregnancies in women with MHVs and modern anticoagulation regimens, VKA is the anticoagulation regimen associated with the lowest risk of adverse maternal outcomes. This finding is consistent with those of previous publications 6, 9, 10, despite the differences in the patients studied; we included many fewer women with ball-in-cage valves and included a study group of women taking dose-adjusted LMWH.

The risk of adverse maternal outcomes with dose-adjusted LMWH throughout pregnancy is higher than that of a VKA regimen. This is balanced by a significant reduction in adverse fetal outcomes. However, on comparison with a subset of individuals who continued taking ≤5 mg of warfarin throughout the duration of pregnancy, no significant difference in fetal risk was observed, thus supporting the notion that warfarin’s teratogenic effects are dose dependent (42). Although the risk of maternal thromboembolic complications remains a concern with LMWH, we report a lower incidence of this complication, compared with previously published meta-analyses 9, 10. The exclusion of fixed-dose LMWH regimens likely accounts for this difference and further strengthens the argument for the use of dose-adjusted LMWH with anti–factor Xa levels during pregnancy. The use of a regimen of LMWH and VKA was not observed to have a lower risk of adverse maternal outcomes, compared with an LMWH regimen, despite the use of a VKA for the majority of each pregnancy. Our data lack granularity with regard to the trimester in which the majority of adverse maternal events occurred, but a possible explanation is that the risk of thrombosis is highest in the first trimester of pregnancy, when VKA risk to the fetus is highest. This could also explain the high incidence of adverse maternal outcomes observed with a regimen of UFH and VKA. It is notable that although the use of a VKA was associated with a high incidence of spontaneous abortion, the incidence of fetal death was quite low, a finding supporting the idea that fetal risk to VKA exposure is highest during early gestation (4). It is also important to recognize that the relative reduction in adverse fetal outcomes with these regimens, compared with VKA, is likely to be significantly underestimated if the comparison warfarin group is limited to individuals who must continue taking >5 mg of warfarin daily to maintain a therapeutic INR, although this comparison was not performed in the current study.

Our results support the American College of Cardiology and American Heart Association guidelines for the management of patients with valvular heart disease, which recommend the use of low-dose warfarin in women who are able to maintain therapeutic INRs (Class IIa) over the use of either first trimester LMWH or UFH use (Class IIb) (7). It is notable that despite findings supportive of these guidelines, only 4 of the referenced publications in the valve guidelines were included in our analysis because of our stringent inclusion and exclusion criteria.

### Study limitations

The data obtained for this meta-analysis were observational, not randomized. Therefore, direct comparisons among regimens using the RARs should be regarded as exploratory and must be interpreted with caution, given the possibility of confounders. For example, the observed differences in adverse maternal and fetal outcomes among anticoagulation regimens may have been influenced by the underlying cardiac disorder (e.g., rheumatic vs. congenital heart disease) that led to valve replacement. Additionally, the data for specific anticoagulation regimens were clustered by region, thus introducing the possibility that differences in access to health care could have influenced outcomes. Second, there is a paucity of published data reporting maternal and fetal outcomes in pregnant women with modern MHVs who are receiving many of the newer anticoagulation regimens, and this scarcity increases the likelihood of a type II error. This may explain the similarity in adverse fetal events between the VKA regimen and the 2 regimens that withhold VKA for the first trimester. Third, our data lack the granularity to determine the trimester in which each maternal event occurred, thereby making it difficult to assess whether individuals receiving heparin in the first trimester and a VKA in the second and third trimesters had a thrombotic event while taking heparin or a VKA. Finally, our composite outcomes do not include maternal hemorrhage, premature delivery, fetal intracranial hemorrhage, and neonatal death because of underreporting and nonuniform definitions of these adverse events. These outcomes, as well as others, are important considerations when determining optimal anticoagulation regimens in this patient population, and they highlight the importance of prospective registries with standardized reporting, such as the ROPAC (Registry of Pregnancy and Cardiac Disease) study of pregnancy in women with MHVs (33).

The significant heterogeneity observed in the estimated risks is likely multifactorial. As mentioned earlier, our analysis included studies from a large geographic distribution and included patients from regions with vastly different access to health care, which may have resulted in greater heterogeneity. Additionally, heterogeneity was greatest for the composite fetal outcome, which was largely driven by the incidence of spontaneous abortions. One explanation for this finding is the apparent dose-dependent effects of VKAs on the developing fetus. It is possible that differences in the distribution of VKA doses among studies were responsible for much of the observed heterogeneity. However, given that most studies included in this group did not report the distribution of VKA doses, we were unable to verify or explicitly model this hypothesis in the meta-regression. The random effects analysis allowed us to account and quantify the heterogeneity among the estimates from the different studies; however, as a result, studies were weighted more uniformly in the analysis. Consequently, smaller, less-precise studies may have had a greater impact on the study results. As such, we performed an analysis using a fixed-effects model to estimate maternal and fetal risk. Similar results were obtained, with the exception that fetal risk in women receiving LMWH and VKA was 8%, one-half of what was estimated using the random effects model. This finding has little impact on our study conclusions and further supports the notion that avoidance of VKAs in the first trimester of pregnancy improves fetal risk.

Despite these limitations, we believe that reporting these data is important to clinical practice because no randomized data currently exist and randomized trials are unlikely, given the complex ethical and social issues involved in choosing an anticoagulation regimen for pregnant women. Therefore, our intention is to consolidate all contemporary primary data relating to this subject, so that practitioners and patients alike gain a greater understanding of the strength of the data that are the basis for current recommendations.

Our meta-analysis consolidates the current experience with up-to-date anticoagulant regimens in contemporary study patients with modern MHVs. Additionally, our study focuses on all 4 anticoagulation regimens for pregnant women with MHVs endorsed by the 2014 American College of Cardiology and American Heart Association valve guidelines (7). Our findings support the current recommendations put forth by the guidelines (Central Illustration). However, our analysis excludes individuals with right-sided MHVs, thus making the results of this study less clinically applicable in this patient population.

**Central Illustration:**
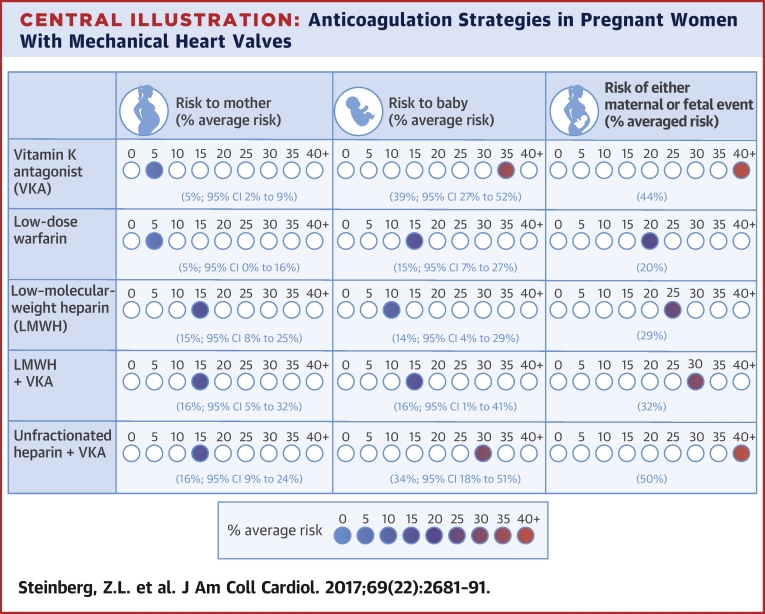
Anticoagulation Strategies in Pregnant Women With Mechanical Heart Valves This illustration depicts maternal and fetal risk with different anticoagulation regimens. Maternal risk is lowest on a vitamin K antagonist (VKA) regimen, and fetal risk is lowest on a low-molecular-weight heparin (LMWH) regimen. The risk of either a maternal or fetal complication during pregnancy is lowest with a low-dose warfarin regimen; however, even low-dose warfarin carries a substantial risk of a poor outcome. CI = confidence interval.

## Conclusions

VKAs represent the safest anticoagulation regimen for pregnant women with mechanical aortic and/or mitral valves. The adverse effects of VKAs on fetal development appear to be limited to early gestation, with low incidences of fetal demise and congenital defects at warfarin doses of ≤5 mg daily. The use of anti–factor Xa–adjusted LMWH, either throughout pregnancy or during the first trimester, followed by warfarin use for the remainder of pregnancy, is associated with higher adverse maternal outcomes as compared with a VKA regimen, but with lower adverse fetal outcomes. However, no difference in adverse fetal outcomes was observed between individuals taking warfarin at doses ≤5 mg daily and those with an LMWH regimen. The use of a regimen of UFH and VKA continues to demonstrate a high risk of adverse maternal outcomes, without a substantially lower risk of adverse fetal outcomes as compared with a VKA regimen. Prospective randomized studies and large patient registry databases are needed to validate these observations.

Perspectives **COMPETENCY IN PATIENT CARE AND PROCEDURAL SKILLS:** In pregnant women with prosthetic MHVs, the use of LMWH as an alternative to VKA anticoagulants results in less fetal loss but a higher incidence of maternal complications. The risk of VKA exposure to the fetus is dose dependent, with relatively low rates of adverse maternal and fetal outcomes with warfarin at doses ≤5 mg daily.

**TRANSLATIONAL OUTLOOK:** The basis of current knowledge of maternal and fetal risks of anticoagulation is a small number of observational studies, and larger, prospective studies are needed to validate these findings.
