# Supplementary material for: Maternal and Fetal Outcomes of Anticoagulation in Pregnant Women With Mechanical Heart Valves
Source: J Am Coll Cardiol. 2017 Jun 6;69(22):2681–91. doi: 10.1016/j.jacc.2017.03.605 (PMC5457289; doi:10.1016/j.jacc.2017.03.605)
Supplement: Online Tables 1–4 [file mmc1.docx]

**ONLINE APPENDIX**

**Online Table 1.** Results from a sensitivity analysis following the exclusion of two studies reporting on combined maternal outcomes for right- and left-sided MHVs (1,2). In the following analysis, 2/41 maternal events were assumed to occur in individuals with left-sided MHVs on a VKA regimen and 0/9 maternal events were assumed to occur in individuals with left-sided MHVs on a UFH + VKA regimen.

| **Composite Maternal Outcome** | | | |
| --- | --- | --- | --- |
| **Regimen** | **Average Risk %** | **Difference of averaged risks** | **Ratio of averaged risks** |
| VKA | 5.1 (2.7, 8.2) |  |  |
| LMWH | 15.2 (7.6, 24.8) | 10 (1, 22) | 3.0 (1.1, 8.1) |
| LMWH+VKA | 15.7 (5.0, 30.7) | 11 (-2, 29) | 3.1 (0.7, 9.4) |
| UFH+VKA | 15.0 (9.1, 22.1) | 10 (2, 19) | 3.0 (1.3, 7.4) |

**Online Table 2.** Results from a sensitivity analysis following the exclusion of one study reporting on combined fetal outcomes for right- and left-sided MHVs (2). This table shows the effect of assuming either 6/9 fetal events or 0/9 fetal events occurred in individuals with left-sided MHVs on a UFH + VKA regimen.

| **Composite Fetal Outcome** | | | |
| --- | --- | --- | --- |
| Assuming 6/9 fetal events occurred in individuals with left-sided MHVs: | | | |
| **Regimen** | **Averaged Risk %** | **Difference of averaged risks** | **Ratio of averaged risks** |
| UFH+VKA | 36.8 (21.7, 53.2) | -2 (-22, 18) | 0.9 (0.5, 1.6) |
| Assuming 0/9 fetal events occurred in individuals with left-sided MHVs: | | | |
| **Regimen** | **Averaged Risk %** | **Difference of averaged risks** | **Ratio of averaged risks** |
| UFH+VKA | 30.4 (16.5, 46.5) | -9 (-28, 11) | 0.8 (0.4, 1.4) |

**Online Table 3.** Results from the mixed effects meta-regression model for the double-arcsine transformed risk of maternal composite outcome comparing cohorts of women who received alternative regimens to women who received VKA, on the double-arcsine transformed risks.

| **Fixed effects** | | **Estimate** | **SE** | **P value** | **95% CI** |
| --- | --- | --- | --- | --- | --- |
|  | Intercept | 0.23 | 0.04 | <0.001 | (0.16, 0.3) |
|  | LMWH | 0.18 | 0.07 | 0.014 | (0.04, 0.32) |
|  | LMWH + VKA | 0.19 | 0.1 | 0.062 | (-0.01, 0.38) |
|  | UFH + VKA | 0.18 | 0.06 | 0.003 | (0.06, 0.31) |
| Between-studies variance (REML) | | 0.008 | 0.005 |  | (0.002, 0.044) |
| I^2^ | | 44% |  |  | (18%, 82%) |

**Online Table 4.** Results from the mixed effects meta-regression model for the double-arcsine transformed risks of fetal composite outcome comparing cohorts of women who received alternative regimens to women who received VKA, on the double-arcsine transformed risks.

| **Fixed effects** | | **Estimate** | **SE** | **P value** | **95% CI** |
| --- | --- | --- | --- | --- | --- |
|  | Intercept | 0.68 | 0.07 | <0.001 | (0.55, 0.81) |
|  | LMWH | -0.29 | 0.11 | 0.012 | (-0.51, -0.06) |
|  | LMWH + VKA | -0.25 | 0.15 | 0.103 | (-0.55, 0.05) |
|  | UFH + VKA | -0.06 | 0.11 | 0.606 | (-0.27, 0.16) |
| Between-studies variance (REML) | | 0.04 | 0.02 |  | (0.02, 0.08) |
| I^2^ | | 81% |  |  | (67%, 90%) |

**References:**

1. Plesinac SD, Darko PV, Pilic IZ, Babovic IR. Anticoagulation therapy during pregnancy of patients with artificial heart valves: fetomaternal outcome. Archives of gynecology and obstetrics 2006;274:141-5.

2. Kawamata K, Neki R, Yamanaka K et al. Risks and pregnancy outcome in women with prosthetic mechanical heart valve replacement. Circ J 2007;71:211-3.
